# Skin deep: Cutaneous manifestation of PIP implant rupture

**DOI:** 10.1016/j.jpra.2021.03.002

**Published:** 2021-03-19

**Authors:** Máire-Caitlín Casey, Edward Jason Kelly

**Affiliations:** Department of Plastic and Reconstructive Surgery, Cork University Hospital, Ireland

**Keywords:** PIP-implant, Implant rupture, Cutaneous manifestation

## Abstract

PIP (Poly Implant Prothèse, France) implants were readily employed for breast reconstruction until withdrawn from the market in 2010. These implants have an early and increased risk of rupture compared to non-PIP implants. This report outlines a significant cutaneous manifestation of PIP-implant rupture not previously described in the literature. This patient developed significant cutaneous xanthomatous inflammation with sinus tract formation that has yet to resolve despite explantation. Further investigation is warranted to elucidate the aetiology of this clinical sign and the optimal management of the cutaneous manifestation.

## Introduction

PIP (Poly Implant Prothèse, France) implants were readily employed for breast reconstruction between the years 2000 and 2010. Due to a significant number of complaints regarding premature rupture received by the *Medicines and Healthcare Products Regulatory Agency* (MHPRA), an investigation into PIP-implants was conducted. The use of unauthorised, industrial-grade silicone and an implant shell of inferior quality were identified, leading to withdrawal of PIP-implants from March 2010. These implants have an early and increased risk of rupture compared to non-PIP implants. Furthermore, the silicone is less cohesive, causing increased local spread following rupture and increased inflammatory response.^1^^,^^2^ While the development of axillary adenopathy and distant silicone granulomata is documented in the literature,^3^, ^4^, ^5^, ^6^ this report outlines a significant cutaneous manifestation of PIP-implant rupture not previously described in the literature.

## Case report

A 68-year-old female presented for review of bilateral breast implants that were inserted in the private sector some 19-years prior. She had been very happy with the breast augmentation however, for the preceding 2-months she developed left breast pain, with localised erythema and oedema over the lower pole of her left breast and inframammary fold (IMF). Past medical history was significant only for hypertension, caesarean section 36years previously and penicillin allergy. She quit smoking 30years prior and was actively working as a carer.

**Figure 1:**
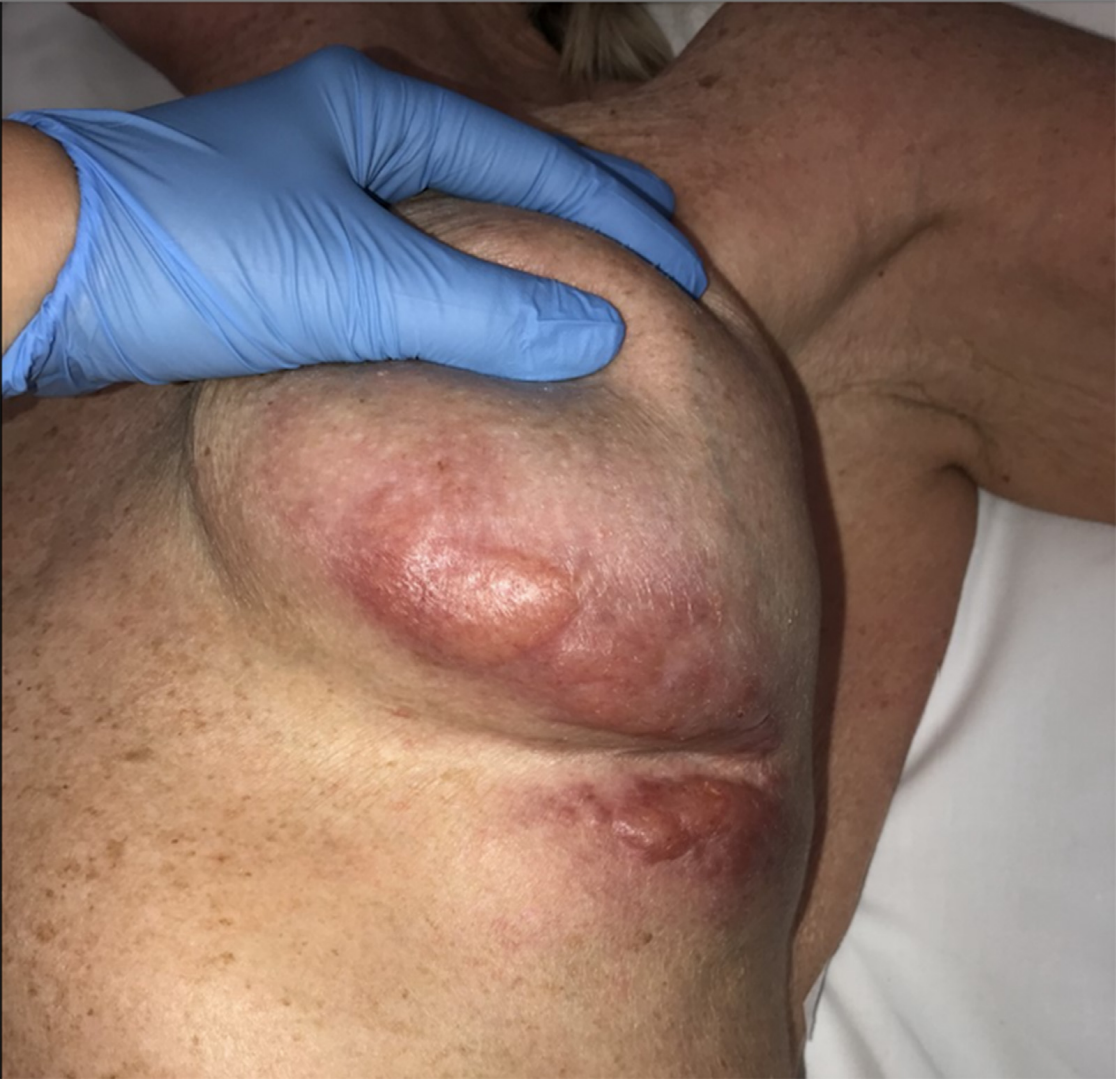
Pre-operative appearance of left breast lower pole and upper abdominal wall.

On examination, bilateral capsular contracture was identified, Baker grade III on the right and grade IV on the left, without adenopathy. On the left breast, below the IMF and on the lower pole of the breast there was a raised, erythematous confluent eruption (Figure 1). To investigate this further, an MRI was arranged, and biopsies performed.

MRI confirmed implant rupture, with extrusion of silicone into the breast and abdominal wall tissue. Biopsies were negative for malignancy, identifying only inflammation.

Following multidisciplinary-team discussion, it was decided to perform bilateral explantation and capsulectomy with expectant management of the skin abnormality.

**Figure 2:**
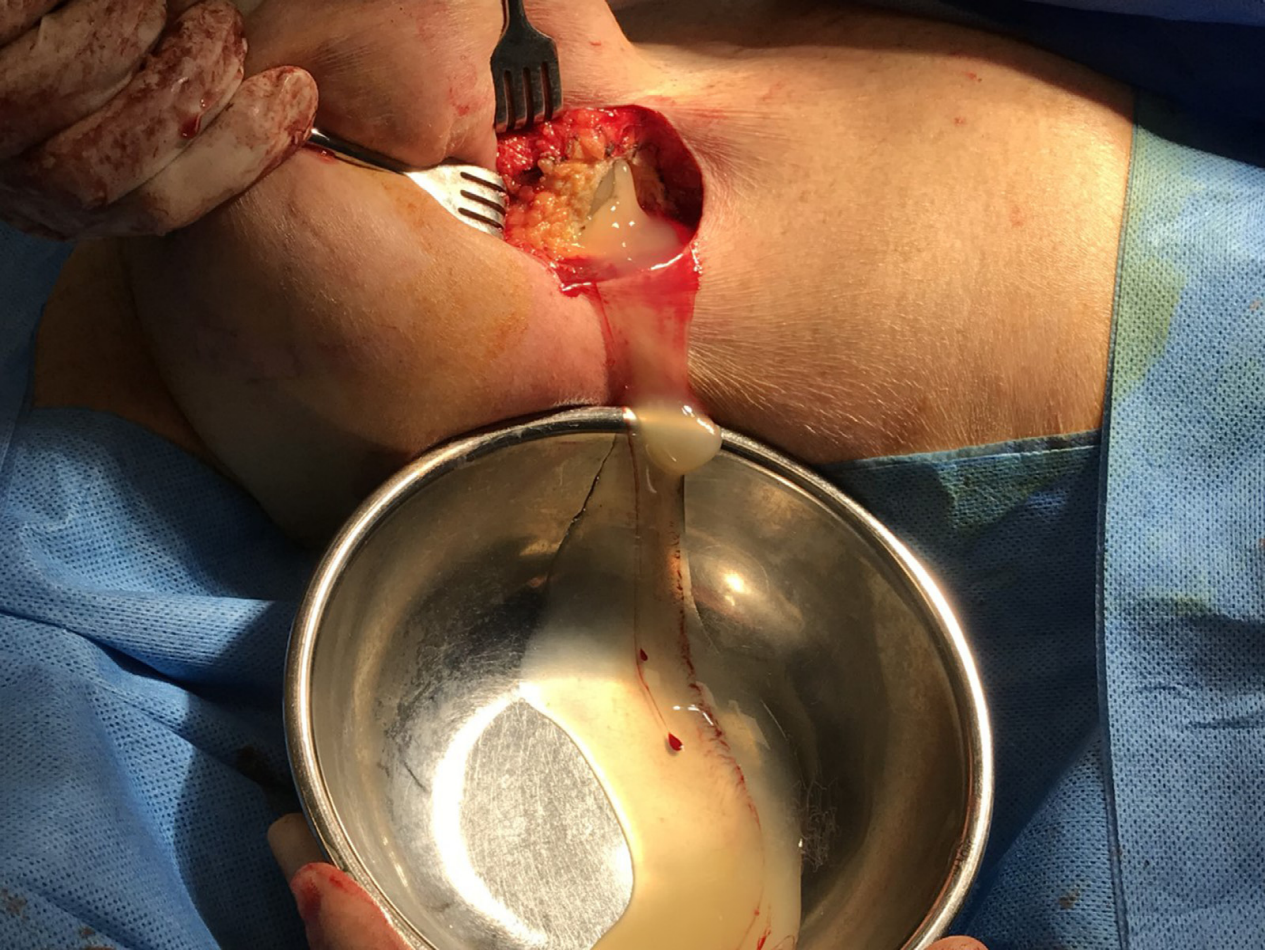
Intra-operative image of right ruptured implant in-situ with extrusion of silicone.

**Figure 3:**
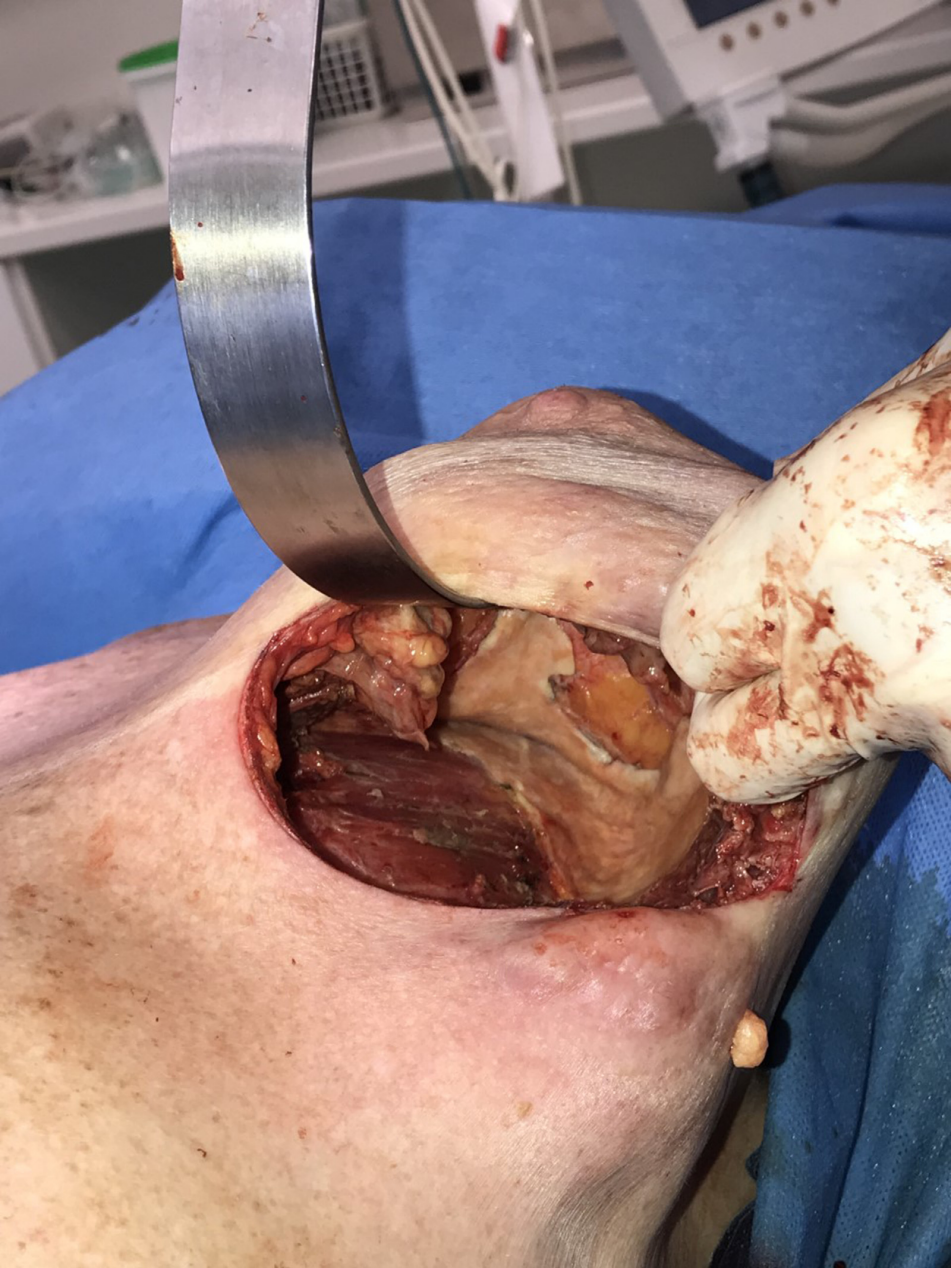
Intra-operative image of left breast cavity post explantation. Tracts of silicone are visible throughout.

Intra-operatively, bilateral ruptured implants were identified. Both cavities were evacuated of copious viscous, yellow silicone material and ruptured silicone shells. Examination of the shells confirmed PIP-implants, size 350cc. Bilateral grossly thickened capsules were visualised, and partial capsulectomies performed, with the capsule material sent for histological analysis (Figure 2). Following copious irrigation, the right-side was closed over a drain. On the left-side, significant and diffuse intraparenchymal silicone was identified (Figure 3). In one area of the left lower pole it was seen to have fistulated through the skin. The skin change was overlying subcutaneous deposits of silicone that were producing what mimicked bullae. Multiple subcutaneous and intraparenchymal serpiginous tracts of silicone required evacuation, prior to copious irrigation and closure with a drain. Further cutaneous biopsies were performed. The patient was kept in hospital overnight and given a short course of antibiotics on discharge, one day post-operatively, following drain removal.

Histological analysis from the capsules identified a ‘*florid extensive organising histiocytic reaction with focally hyalinised and calcified capsule, in keeping with rupture’*. There was no evidence of malignancy or breast implant-associated anaplastic large cell lymphoma (BI-ALCL). The cutaneous biopsies identified only xanthomatous inflammation.

**Figure 4:**
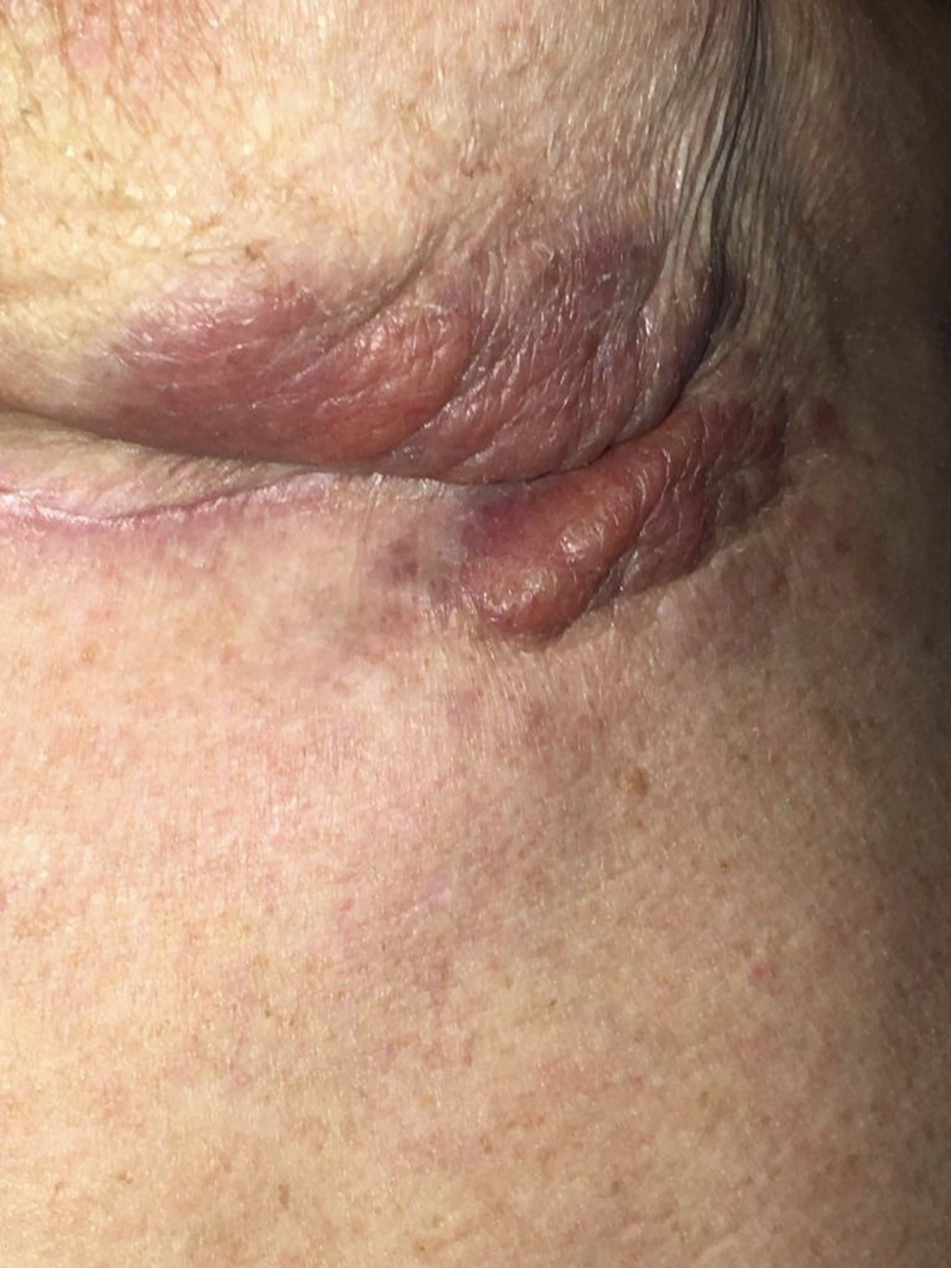
Three months post-operatively.

Three-months post-operatively, the patient was doing well. The cutaneous findings were somewhat improved, and she had no discomfort. Conservative management of this skin reaction is still being pursued (Figure 4).

## Discussion

This patient had known PIP-implants inserted 19-years prior to presentation with explanted silicone shells confirming this. In the setting of breast-implant rupture, further to development of axillary lymphadenopathy, the systemic dissemination of silicone is readily documented in the literature, with reports of silicone granulomata in the upper limbs, the lower limbs, intrapulmonary and abdominal wall.^3^, ^4^, ^5^, ^6^ This case report outlines extensive parenchymal involvement and cutaneous xanthomatous inflammation in the setting of PIP implant rupture that has not previously been described.

A skin rash distant to the site of PIP-implant rupture was reported by Cawrse and Pickford.^7^ Reddish papules were identified on the dorsal hand, forearm and thigh, with biopsies confirming granulomatous inflammation that was attributed to the ruptured breast implant. Mallon, Ganachaud^8^ outlined a case of PIP-implant rupture that presented with only an erythematous rash over the trunk, that extended to affect the breast mounds. This resolved within 3-weeks of explantation. Analysis of the PIP implant composition revealed significant differences bilaterally. This current case-report outlines bilateral rupture with only one breast being affected with cutaneous signs. This raises the query as to whether the variable silicone composition of PIP implants in particular could possibly be responsible for the diverse clinical manifestations following rupture. The development of a maculopapular rash has also be reported in association with non-PIP implant rupture.^9^ However, as cutaneous manifestations of silicone-implant rupture are scarcely reported in the literature, further investigation is warranted.

While infiltration of the breast parenchyma with silicone, secondary to implant rupture, is documented in the literature, it is important to outline the extent of granuloma formation in this patient. Extensive serpiginous tracts of silicone were present throughout the breast parenchyma and subcutaneously in the lower pole of the breast and below the IMF onto the upper abdominal wall with skin fistulation. The cutaneous involvement affected two sites; the lower pole of the breast laterally, 9 × 3 cm, and below the IMF, 5 × 2 cm (Figure 1). This posed a challenging management problem. Excising the cutaneous involvement would result in significant deformation, requiring reconstruction. It was therefore decided to evacuate all silicone with the expectation that the cutaneous derangement would resolve with removal of the causative agent. While full resolution has not occurred, mild improvement is visible.

To date, routine explantation of PIP implants is not recommended as there is insufficient data to support associated cytotoxicity or genotoxicity.^2^^,^^10^ In cases of confirmed rupture however, explantation is recommended.

## Conclusion

Cutaneous manifestations of silicone implant rupture, although rare, can be quite disfiguring. Further investigation is warranted to elucidate the aetiology of this clinical sign and the optimal management of the cutaneous manifestation.

## Consent

I have obtained informed consent from the patient involved to use her case details and clinical photographs for publication.

## Declaration of Competing Interest

Nil to disclose.

## References

[1] Lahiri A., Waters R. (2006). Locoregional silicone spread after high cohesive gel silicone implant rupture. J Plast Reconstr Aesthet Surg.

[2] (SCENIHR) SCoEaNIHR. The safety of Poly Implant Prothèse (PIP) silicone breast implants. Update of the opinion of February 2012. 2014.

[3] Capozzi A., Du Bou R., Pennisi V.R (1978). Distant migration of silicone gel from a ruptured breast implant. Case report. Plast Reconstr Surg.

[4] Dragu A., Theegarten D., Bach A.D., Polykandriotis E., Arkudas A., Kneser U. (2009). Intrapulmonary and cutaneous siliconomas after silent silicone breast implant failure. Breast J.

[5] Ahn C.Y., Shaw W.W. (1994). Regional silicone-gel migration in patients with ruptured implants. Ann Plast Surg.

[6] Oh J.H., Song S.Y., Lew D.H., Lee D.W. (2016). Distant migration of multiple siliconomas in lower extremities following breast implant rupture: case report. Plast Reconstr Surg Glob Open.

[7] Cawrse N.H., Pickford M.A. (2011). Cutaneous manifestation of silicone dissemination from a PIP implant – a case for prophylactic explantation?. J Plast Reconstr Aesthet Surg.

[8] Mallon P., Ganachaud F., Malhaire C., Brunel R., Sigal-Zafrani B., Feron J.G. (2013). Bilateral poly implant prothese implant rupture: an uncommon presentation. Plast Reconstr Surg Glob Open.

[9] Koh E., Watson D.I., Dean N.R. (2016). An uncommon presentation of breast implant rupture. Plast Reconstr Surg Glob Open.

[10] Keogh S.B. Poly Implant Prothèse (PIP) breast implants: final report of the Expert Group. 2012.

